# Garlic Attenuates Plasma and Kidney ACE-1 and AngII Modulations in Early Streptozotocin-Induced Diabetic Rats: Renal Clearance and Blood Pressure Implications

**DOI:** 10.1155/2016/8142394

**Published:** 2016-05-18

**Authors:** Khaled K. Al-Qattan, Martha Thomson, Divya Jayasree, Muslim Ali

**Affiliations:** Department of Biological Sciences, Faculty of Science, Kuwait University, P.O. Box 5969, 13060 Safat, Kuwait

## Abstract

Raw garlic aqueous extract (GE) has ameliorative actions on the renin-angiotensin system in type-1 diabetes mellitus (DM); however its effects on plasma and kidney angiotensin I converting enzyme type-1 (ACE-1) and angiotensin II (AngII) require further elucidation. This study investigated the effect of GE on plasma and kidney ACE-1 and AngII concentrations and in relation to systemic and renal clearance indicators significant to blood pressure (BP) homeostasis in early streptozotocin- (STZ-) induced type-1 DM. Normal rats (*n* = 10) received 0.5 mL normal saline (NR/NS), diabetic rats (*n* = 10) received 0.5 mL NS (DR/NS), and treated diabetic rats (*n* = 10) received 50 mg/0.1 mL/100 g body weight GE (DR/GE) as daily intraperitoneal injections for 8 weeks. Compared to NR/NS, DR/NS showed a significant increase in plasma ACE-1 and AngII and conversely a decrease in kidney ACE-1 and AngII. These changes were associated with an increase in BP and clearance functions. Alternatively and compared to DR/NS, DR/GE showed normalization or attenuation in plasma and kidney ACE-1 and AngII. These GE induced rectifications were associated with moderation in BP elevation and renal clearance functions. Garlic attenuates modulations in plasma and kidney ACE-1 and AngII, in addition to BP and renal clearance function in type-1 DM.

## 1. Introduction

The endocrinal renin-angiotensin system (RAS) was initially described as follows: upon stimulation, renin, a protease, is released by both kidneys to the general circulation. In the plasma, renin acts on angiotensinogen, an *α*-globulin synthesized by the liver, to liberate a decapeptide known as angiotensin I (AngI). While passing through the pulmonary circulation, AngI is cleaved by a dipeptidyl dipeptidase known as angiotensin I converting enzyme (ACE-1) to free an octapeptide called angiotensin II (AngII) [1]. Recently, it has been asserted that the kidneys produce all components of the RAS [2, 3].

ACE-1 is the second rate limiting enzyme that controls the liberation of AngII: the most active component of the RAS [4]. AngII has numerous biological activities, including vasoconstriction, antinatriuresis, and antidiuresis, actions which are closely affiliated with renal clearance functions and BP regulation [5]. It is most probable that under physiological conditions renally and systemically produced AngII work synergistically, where renal AngII acts as the principle paracrine regulator of the kidneys' clearance function determinants, including renal hemodynamics, glomerular filtration rate (GFR), and tubular handling of electrolytes and water [6]. Alternatively, systemically produced AngII operates as the main telocrine modulator of, firstly, general and, secondly, peripheral vascular resistance [7]. The fine tuning of the AngII-mediated actions is achieved by AngII binding to and activating either/or both of its two major receptor types: AT_1_ and AT_2_ [8, 9]. Specifically, AT_1_ receptor facilitates the AngII known actions of general and peripheral vasoconstriction, renal antinatriuresis and antidiuresis, and cell growth and proliferation [10], while the AT_2_ receptor mediates the suggested AngII vasodilatation, natriuresis and diuresis, apoptosis, and antiproliferation, actions that antagonize those evoked by the AT_1_ receptor type [11].

Dysregulation in the RAS associated with AngII AT_1_/AT_2_ receptors expression imbalance is major factors in the initiation and progression of tissue remodeling and refunctioning in biochemical-physiological pathologies [8, 10, 12, 13] including diabetes mellitus (DM) [14]. DM is a progressive disease that entails dynamic phase-changing, structural-functional characteristics. In an attempt to elucidate the nature of RAS modulations in insulin-dependent type-1 DM [15], the findings of previous studies led to the formulation of two major conflicting views.

A group of studies have suggested an increase in the RAS activity and consequently ACE-1 and AngII bioavailability especially in renal tissue and nephronal structures. This view was supported by findings that inhibitors of different RAS components and AngII receptor blockers were effective in partially diminishing DM abnormalities [12, 14]. An augmented RAS activity most likely occurs at later stages of DM when molecular/cellular transformations collectively lead to severe renal-nephronal injuries; in particular glomerulotubular sclerosis, which impedes kidney clearance function leading to the end-stage renal failure that is characteristic of advanced DM [16–18]. A hypoinsulinemic-hyperglycemic provoked high AngII concentration and stimulation of overexpressed AT_1_ receptors lead to the following events in the kidneys: sodium transport and retention, vascular resistance, glomerular capillary pressure, mechano-stretch-induced reactive oxygen species production, and tubulointerstitial cell hypertrophy and hyperplasia associated with extracellular mesangial matrix production [19, 20]. This AngII/AT_1_ scenario is exacerbated by downregulation of intrarenal AT_2_ receptor expression [21] and subsequent minimization of its mediated alleviating responses including inhibition of Na^+^-K^+^-ATPase activity [22], sodium pump action in renal proximal tubules [23], antinatriuresis [24], vasoconstriction [11], cell hypertrophy, and renal glomerular and tubular remodeling [8, 10, 25].

Other studies have reported opposite findings and variations in the activity of plasma and renal RAS components, particularly ACE-1 and AngII, in early DM. In 4 weeks after streptozotocin- (STZ-) induced type-1 DM rats, it was reported that the RAS is downregulated at the level of mRNA expression [26]. Furthermore, renal ACE content [27] as well as AngII concentration [28] was reduced. In addition, a further decline in AngII concentration was suggested to result from an increase in degradation by the enzyme angiotensinase A [29]. In a review, Copper et al. [30] argued that RAS activation is controversial as different animal-molecular studies reported conflicting results. In particular, it was suggested that in early DM the indices of RAS are lower and the concentrations of renal AngII and its receptor AT_1_ are reduced leading to hyperfiltration. An increased GFR, which can be monitored by measuring creatinine clearance in live subjects [31], with a suggested lower AngII type AT_1_ receptor-mediated activation of sodium retention leads to higher sodium and water clearance. This excretory behavior causes polyuria, in addition to albuminuria, and necessary, however futile, polydipsia: a myriad of symptoms that are typically observed at the early stages of DM.

Garlic has long been used in traditional medicine as an easily available and accessible natural medicine [32] to control BP and sugar in general [33] and when affiliated with DM complications [34]. Within the past two decades, garlic, either as an aqueous extract (GE) or isolated organosulfur constituents, has been the focus of intensive studies in the STZ-induced experimental model of type-1 DM [35] showing several interesting and well documented ameliorative actions [36]. Among these actions is garlic's ability to induce several biochemical-physiological measures at the systemic level; particularly, serum insulin elevation [37, 38], blood glucose reduction [38, 39], serum ACE activity diminution and suggested reduced AngII generation [40], and, therefore, hypotension [36]. The induced hypotension may be mediated, in part, through a reduced AngII concentration resulting in lower vasoconstrictive action and/or indirectly through an increase in the amounts and actions of vasodilatory agents [41]. More recently, our group has also shown that GE treatment in early STZ-DM improved kidney clearance functions [42], in addition to preserving the normal expression and balance of the two AngII receptors types [43, 44].

To further elucidate garlic's ameliorative mechanism related to RAS in early type-1 DM, this study investigated the effect of GE on DM-induced changes in plasma and renal ACE-1 and AngII concentrations. The effects of GE on ACE-1 and AngII were correlated to simultaneous modulations in BP and systemic and renal indicators of clearance function.

## 2. Materials and Methods

### 2.1. Materials

The materials used in this study, unless otherwise stated, were obtained accordingly: analysis kits as indicated in this section, Thiopental Sodium from May & Baker (England) and STZ, chemicals, and reagents from Sigma-Aldrich (USA).

### 2.2. Preparation of Raw Garlic Aqueous Extract

The GE (50 mg/0.1 mL) used in this study was prepared from locally purchased, peeled fresh garlic cloves (*Allium sativum L.*) as previously described [45]. The prepared GE was immediately stored as 1 mL aliquots in 1.5 mL self-capping, inert-plastic freeze-durable Eppendorf tubes at −20°C. The required volume of GE was thawed daily to ambient temperature before administration and following use any remaining amount was discarded. GC-MS comparative analysis of the GE after different times of freeze-storage showed similar composition and concentration of components [42, unpublished data].

### 2.3. Animals

The animals used in this study were male Sprague-Dawley rats (ancestors origin: Harland Lab, Oxfordshire, England) having an initial body weight of 150–200 grams. Before and during the study, the rats were kept in adequate-size separate cages and housed in an Animal Care Facility under standard ambient conditions (23 ± 2°C, natural day/night cycle). The rats were provided with standard rodent diet (170 mmol Na^+^/kg) and tap water* ad libitum*.

The care and use of all rats used in this study was in full compliance with the Guide for the Care and Use of Laboratory Animals, National Research Council [46].

### 2.4. Induction of Type-1 Diabetes

The type-1 DM rats used in this study were produced by intraperitoneally injecting each of a sufficient number of overnight fasting rats with a single dose of STZ (6 mg/100 g body weight) dissolved in citrate buffer (0.3 mL, 0.01 M, pH 4.5) as described previously [39]. After 5 days, STZ-injected rats with a blood glucose concentration ≥16 mmol/L measured in a drop of tail-blood (One Touch UltraEasy Glucometer, UK) under mild ether sedation were deemed diabetic (*n* = 20) and used in the study.

### 2.5. Rats' Groups and Treatments

At day 7 after STZ injection, DM rats were divided into two groups and treated for 8 weeks with either a single daily intraperitoneal injection of 0.1 mL of normal saline/100-gram body weight (DR/NS, *n* = 10) or 50 mg/100-gram body weight/0.1 mL of GE (DR/GE, *n* = 10). For reference, normal rats, injected initially with 0.3 mL of only citrate buffer and having normal blood glucose ≤8 mmol/L (*n* = 10), received a single daily intraperitoneal injection of 0.1 mL/100-gram body weight of normal saline (NR/NS, *n* = 10) also for 8 weeks.

### 2.6. Measurements of Blood Glucose, Blood Pressure, Water Intake, and Urine Output

The following parameters were measured for all rats in each group as follows: blood glucose before and at weeks 4 and 8 of respective treatment; BP at weeks 1 and 8 of respective treatment as an average of 3 readings for each rat using the tail-cuff method (Harvard Apparatus, England); water intake and urine output before and at weeks 1, 4, and 8 of respective treatment for 24 h and calculated for 1 h.

### 2.7. Collection of Blood and Preparation of Plasma and Serum Samples

At the end of the 8-week treatment period, each rat was anesthetized with an intraperitoneal injection of Thiopental Sodium (4–6 mg/100 g). Within 2-3 minutes, blood was collected via cardiac puncture from each rat as 3 separate portions of 2 mL each into 3 × 15 mL inert-plastic tubes (Falcon, USA) and treated accordingly: (1) 2 mL blood in a tube containing 0.4 mL of a peptidase inhibitor cocktail (0.2 mL trisodium citrate (0.1 M), 0.05 mL O-phenanthroline (0.44 mM), 0.05 mL pepstatin (0.12 mM), 0.05 mL EDTA (0.6 M), and 0.05 mL P-hydroxymercuribenzoic acid (1 mM)) for plasma preparation used for AngII concentration determination, which was done immediately as described below; (2) 2 mL blood in a tube containing 0.2 mL EDTA for plasma preparation used for ACE-1 concentration estimation; (3) 2 mL blood in a tube for serum collection used for insulin, albumin, and creatinine measurement. Collected plasma (except for AngII analysis) and serum samples were stored as approximately 0.5 mL aliquots in Eppendorf tubes at −40°C for later analysis.

### 2.8. Preparation of Kidneys' Homogenate and Collection of Supernatant Samples

Following collection of blood and within 30–45 seconds, the left kidney of each rat was excised and while bathing in the peptidase inhibitor cocktail decapsulated cut into 4-5 portions and placed separately in 10 mL, capped inert-glass vials containing 3 mL of the inhibitor. Also, within 30–45 seconds, the right kidney was excised and while bathing in Tris-HCl (0.05 M, pH = 7.6) buffer decapsulated, cut into 4-5 portions, and placed separately in a 10 mL vial containing 3 mL of the buffer. Afterwards, each kidney was homogenized, allowed to stand on ice for few minutes, and then centrifuged for 15 minutes at 8000 ×g at 4°C. The supernatant of each right kidney was stored separately as 0.5 mL aliquots in Eppendorf tubes at –40°C for later analysis, while the supernatant of the left kidney was assayed immediately for AngII and protein concentrations as described below.

### 2.9. Determination of Insulin, Angiotensin Converting Enzyme I, Angiotensin II, Albumin, and Creatinine Concentrations

These parameters were quantitated using analysis kits supplied as indicated and following the manufacturers' instructions: serum insulin was measured by the ELISA method using kits from SPIbio (France); plasma and kidney ACE-1 concentration were also determined by the ELISA method using kits acquired from Uscn Life Sciences Inc. (China); plasma and kidney AngII were measured by the immunoassay procedure using kits from RayBiotech (USA); serum and urine albumin were determined by a colorimetric technique using kits from BioAssay Systems (USA); and finally, serum and urine creatinine levels were estimated by a colorimetric method using kits from Randox (USA).

### 2.10. Number of Experimentation Cycles Carried Out

All procedures stated above were carried out through the necessary number of experimental cycles to replace rats that did not develop typical symptoms of DM after STZ injection or died during the different treatment protocols. The rate of success of the first cycle was very high.

### 2.11. Statistical Analysis

The data are presented in bar graphs as the mean ± SEM of the absolute values of the measured and calculated parameters. Statistical differences between the 3 groups were calculated using* One-Way ANOVA* (SPSS, V 22, IBM) with LSD post hoc test at *P* < 0.05 indicating significance. Differences between the 3 groups at each corresponding stage of the experiment are also presented in percentage values in Results.

## 3. Results

### 3.1. Effect of Garlic Extract on Blood Glucose, Body Weight, Water Intake, Urine Output, and Blood Pressure

**Figure 1:**
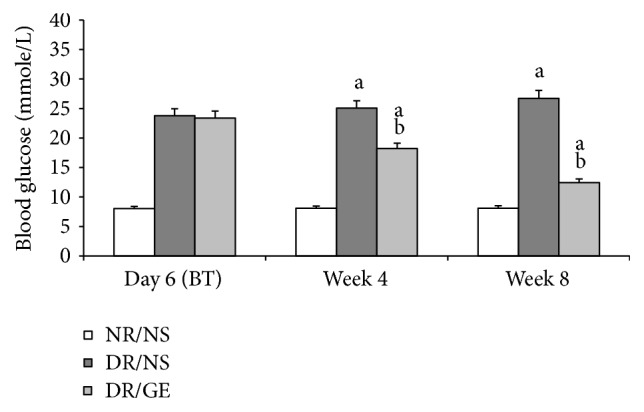
GE treatment decreased blood glucose of diabetic rats. Blood glucose was measured at day 6 after STZ injection and at the end of weeks 4 and 8 of the treatment period. NR/NS: normal rats/normal saline treated; DR/NS: diabetic rats/normal saline treated; DR/GE: diabetic rats/garlic extract treated; BT: before treatment; ^a^significantly different compared to NR/NS; ^b^significantly different compared to DR/NS.

At day 6 after STZ injection and before commencing the treatment protocol, the blood glucose level of both diabetic rats groups was significantly higher by 195% compared to that of the NR/NS group (Figure 1). The elevated blood glucose was sustained in the DR/NS group during the 8-week treatment period. Alternatively, at week 4 and week 8 of treatment, the DR/GE group had blood glucose levels that were significantly less by 27 and 54%, respectively, compared to the DR/NS group; however, these levels were significantly higher than those of NR-NS group by 128% at week 4 and 55% at week 8 (Figure 1).

**Figure 2:**
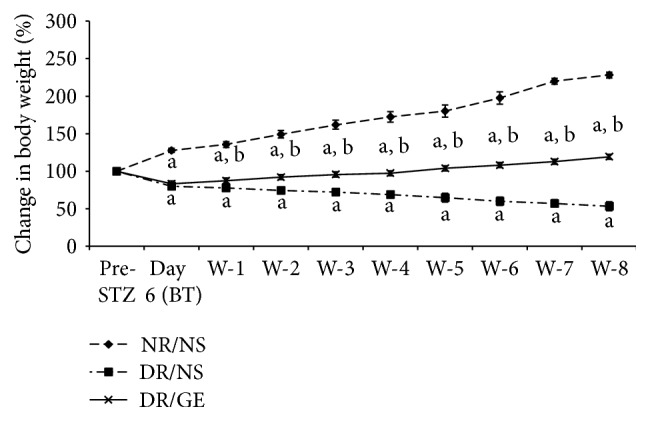
GE treatment increased body weight of diabetic rats. GE was administered IP to diabetic rats for 8 weeks abbreviated at DR/GE. Diabetic control rats (DR/NS) and normal control rats (NR/NS) were given normal saline. The animals were weighed before STZ injection (pre-STZ), 6 days after STZ injection (day 6 (BT)), and weekly for the 8-week treatment period. Weights are plotted as percentiles with the starting weights all standardized to 100%. Pre-STZ: before streptozotocin administration; BT: before treatment; w: week; ^a^significantly different compared to NR/NS; ^b^significantly different compared to DR/NS.

The initial body weight average of the rats for each group was similar among the 3 groups. However, as the study proceeded, the average body weight started to shift at day 6 after STZ injection, where the NR/NS body weight started to increase, while both diabetic groups' body weight started, slightly but significantly, to decline compared to their and the NR/NS initial weight. As the treatment proceeded, the NR/NS weight increased steadily, while the DR/NS weight decreased continuously. Alternatively, the DR/GE weight picked up from the initial drop and even showed a slight gain at the last 2-3 weeks compared to their starting weight. At the end of the treatment protocol, the 3 groups of rats showed the following changes in body weight: NR/NS gained 135%; DR/NS lost 54%; DR/GE gained 18% (Figure 2).

**Figure 3:**
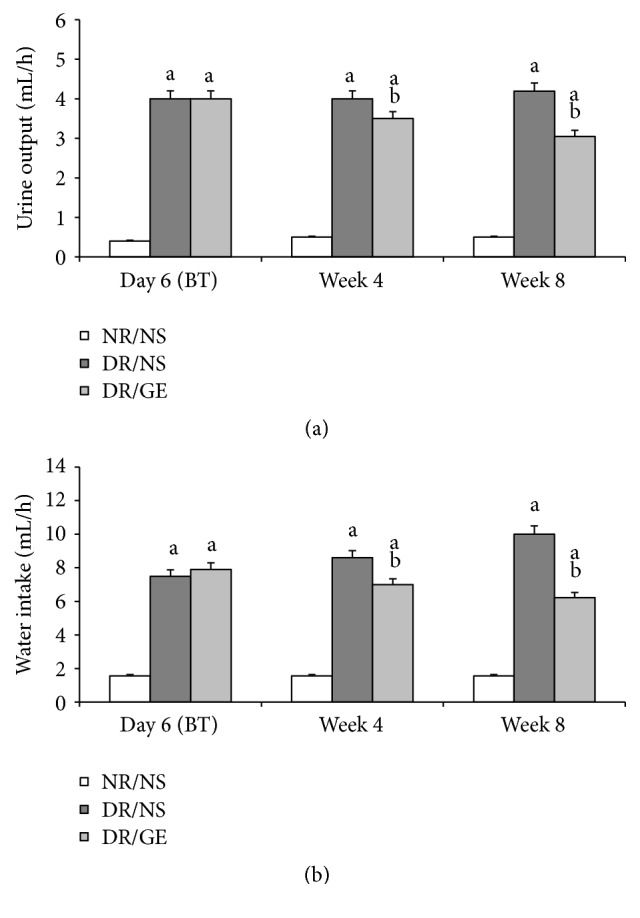
GE treatment decreased urine output and water intake of diabetic rats. (a) Urine output (mL/h) and (b) water intake (mL/h) were measured before treatment (BT) and after 4 and 8 weeks of treatment. NR/NS: normal rats/normal saline treated; DR/NS: diabetic rats/normal saline treated; DR/GE: diabetic rats/garlic extract treated; BT: before treatment; ^a^significantly different compared to NR/NS; ^b^significantly different compared to DR/NS.

Again, at day 6 after STZ injection and before starting the treatment protocol, the measured water intake and urine output of both diabetic groups were significantly higher by an average of 381 and 900%, respectively, compared to the water intake and urine output measured for the NR/NS group. The NR/NS group's water intake and urine output did not change significantly when measured at week 4 and week 8. As far as the DR/NS group is concerned, not only did these rats' elevated water intake and urine output remain significantly higher, but their water intake even increased steadily to higher levels at week 4 and week 8 of the treatment period. As for the DR/GE group, although these rats water intake and urine output were still significantly higher than the measured values of the NR/NS at week 4 and week 8 by an average of 323 and 555%, respectively, the values of these parameters at these same weeks for the GE-treated diabetic group were significantly less by an average of 29 and 20%, respectively, compared to the values of the DR/NS group (Figure 3).

**Figure 4:**
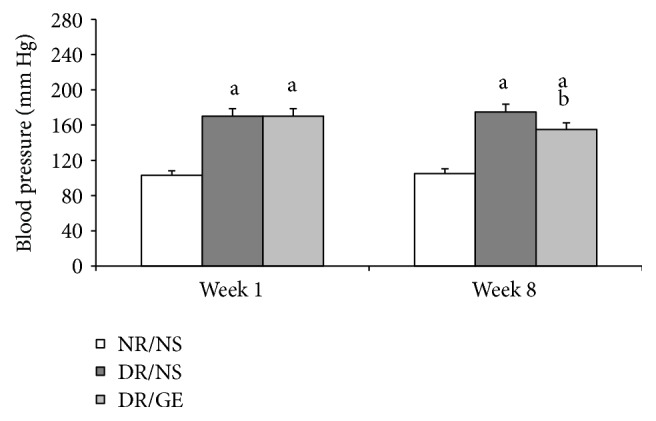
GE treatment lowers blood pressure of diabetic rats. Systolic blood pressure (mm Hg) was measured after STZ (week 1) and at the end of the treatment period (week 8). NR/NS: normal rats/normal saline treated; DR/NS: diabetic rats/normal saline treated; DR/GE: diabetic rats/garlic extract treated; ^a^significantly different compared to NR/NS; ^b^significantly different compared to DR/NS.

At week 1 of the treatment period, the NR/NS group BP was within normal range and remained at that level when measured at week 8. Alternatively and at week 1, both the DR/NS and DR/GE groups had a significantly higher BP by an average of 65% compared to the NR/NS group. Although this elevated BP was still evident for both of the DR/NS and DR/GE groups at week 8, it was slightly but significantly less in the DR/GE group by an average of 10% compared to both this group's reading at week 1 and the DR/NS group reading at week 8 (Figure 4).

### 3.2. Effect of Garlic Extract on Insulin, Angiotensin Converting Enzyme-1, Angiotensin II, Albumin, and Creatinine

**Figure 5:**
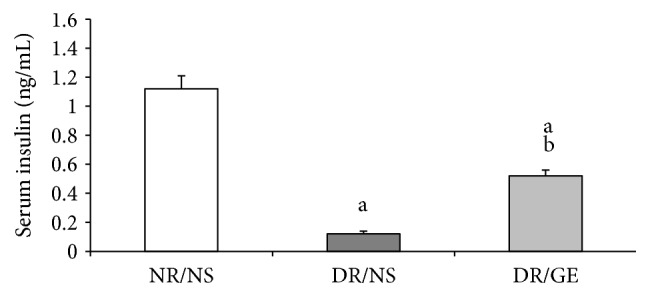
GE treatment increased serum insulin of diabetic rats. Serum insulin was quantitated at the end of the treatment period (week 8). NR/NS: normal rats/normal saline treated; DR/NS: diabetic rats/normal saline treated; DR/GE: diabetic rats/garlic extract treated; ^a^significantly different compared to NR/NS; ^b^significantly different compared to DR/NS.

At the end of the 8-week treatment period, the serum insulin level for the DR/NS group was significantly less by 89% compared to the insulin value measured for the NR/NS group. In the DR/GE group, although the insulin level was still less than for the NR/NS by 54%, it was significantly higher by 331% compared to the insulin level measured for the DR/NS group (Figure 5).

**Figure 6:**
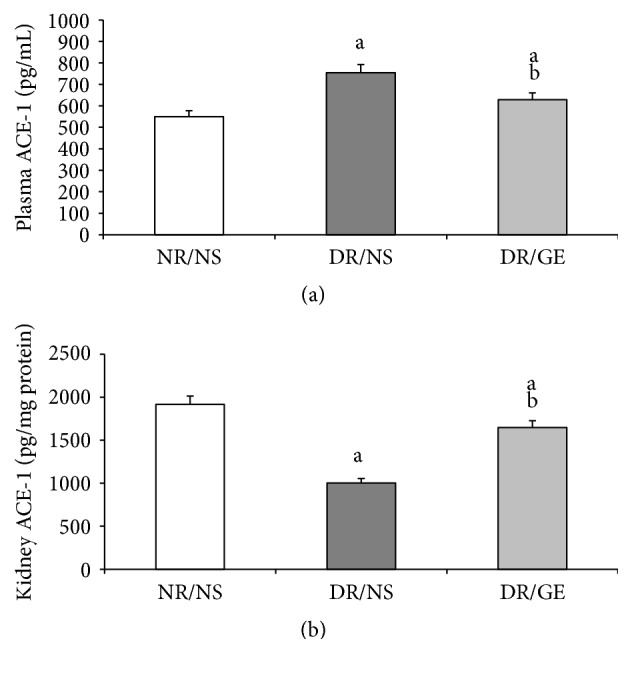
GE treatment decreased plasma ACE-1 and increased kidney ACE-1 in diabetic rats. ACE-1 was quantitated in both (a) plasma (pg/mL) and (b) kidney (pg/mg) at the end of the treatment period (week 8). NR/NS: normal rats/normal saline treated; DR/NS: diabetic rats/normal saline treated; DR/GE: diabetic rats/garlic extract treated; ^a^significantly different compared to NR/NS; ^b^significantly different compared to DR/NS.

The plasma and kidney levels of ACE-1 in the NR/NS group were 550 pg/mL and 1917 pg/mg protein, respectively. In the DR/NS group, and compared to the NR/NS, ACE-1 levels showed opposite, yet significant changes, where the plasma ACE-1 was higher by 37% and the kidney ACE-1 was lower by 48%. In the DR/GE group, the plasma and kidney ACE-1 values, with only minor but significant difference of 14%, were almost comparable to those measured in the NR/NS group (Figure 6).

**Figure 7:**
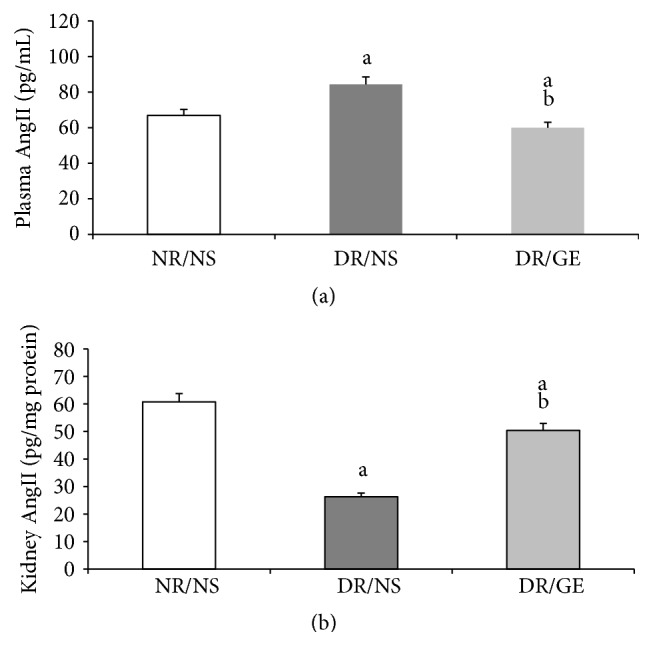
GE treatment decreased plasma AngII and increased kidney AngII in diabetic rats. AngII was quantitated in both (a) plasma (pg/mL) and (b) kidney (pg/mg) at the end of the treatment period (week 8). NR/NS: normal rats/normal saline treated; DR/NS: diabetic rats/normal saline treated; DR/GE: diabetic rats/garlic extract treated; ^a^significantly different compared to NR/NS; ^b^significantly different compared to DR/NS.

In the DR/NS and DR/GE rats, the observed changes in the plasma and kidney AngII levels showed parallel behaviors to those quantitated for their respective ACE-1 concentrations. More precisely, the plasma AngII level was significantly higher by 25% in the DR/NS group compared to that in the NR/NS group and less by 29% in the DR/GE group than in the DR/NS group. As for the kidney AngII concentration, it was significantly less by 57% in the DR/NS group than in the NR/NS group and higher by 92% in the DR/GE group than in the DR/NS group. Although the plasma and kidney AngII levels in the DR/GE group were almost comparable to those in the NR/NS, they were still significantly different by 10 and 22%, respectively, in a manner almost similar to that observed for the ACE-1 (Figure 7).

**Figure 8:**
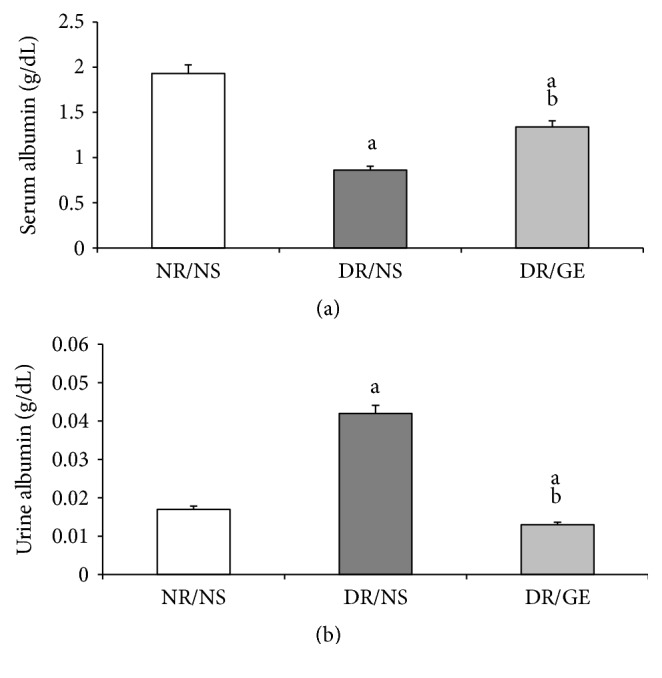
GE treatment reversed albuminuria of diabetic rats. Serum albumin (a) and urine albumin (b) were determined (g/dL) at the end of the treatment period (week 8). NR/NS: normal rats/normal saline treated; DR/NS: diabetic rats/normal saline treated; DR/GE: diabetic rats/garlic extract treated; ^a^significantly different compared to NR/NS; ^b^significantly different compared to DR/NS.

The concentration of serum albumin was significantly less by 55% in the DR/NS group than in the NR/NS group. On the other hand, in the DR/GE group, the level of serum albumin was significantly higher by 56% than in the DR/NS group and less by 31% compared to that in the NR/NS group. The urine albumin showed opposite concentration patterns to those measured for the serum in the three rat groups. The level of urine albumin was considerably higher by 147% in the DR/NS group compared to the NR/NS group and was less by 69% in the DR/GE group than in the DR/NS group, which was less by 24% than in the NR/NS group (Figure 8).

**Figure 9:**
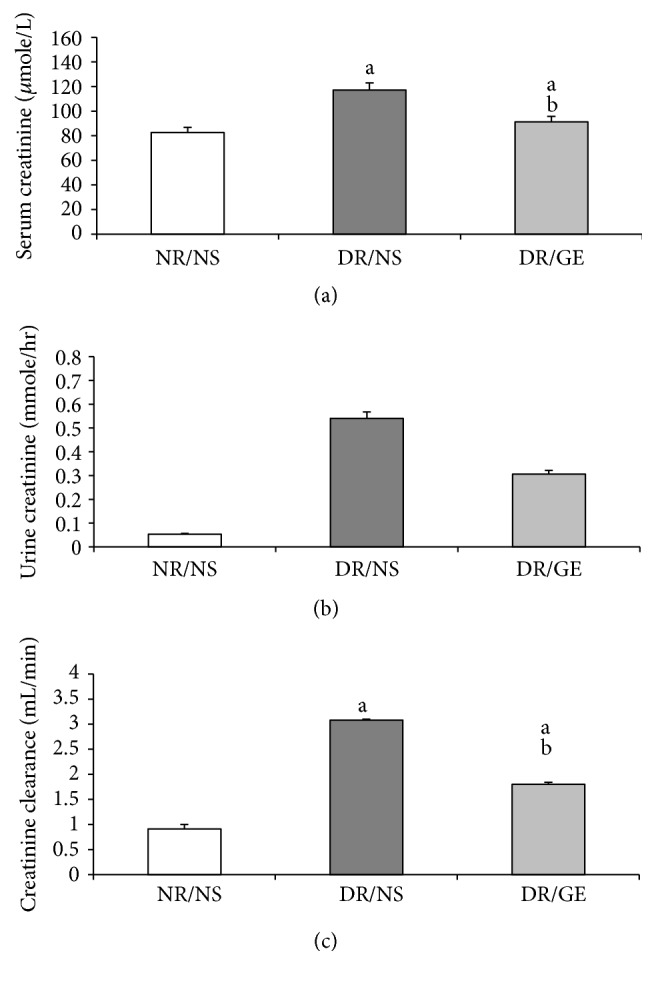
GE treatment ameliorated changes in creatinine levels and creatinine clearance of diabetic rats. Creatinine levels in (a) serum (*μ*mole/L) and (b) urine (mmole/hr) were determined at the end of the treatment period (week 8). (c) Creatinine clearance (mL/min) was calculated from these values. NR/NS: normal rats/normal saline treated; DR/NS: diabetic rats/normal saline treated; DR/GE: diabetic rats/garlic extract treated; ^a^significantly different compared to NR/NS; ^b^significantly different compared to DR/NS.

The magnitude of serum creatinine was significantly higher by 41% in the DR/NS group compared to the NR/NS group, while in the DR/GE group the serum creatinine level was significantly less by 22% than in the DR/NS group and higher by 9.6% compared to that measured in the NR/NS group (Figure 9(a)). The urine creatinine levels showed similar pattern of change to that observed in the serum of the diabetic groups. Notably, the urine creatinine level was considerably higher by 908% in the DR/NS group compared to that quantified for the NR/NS group and less by 43% in the DR/GE group compared to that in the DR/NS group. Furthermore, the urine creatinine level remained higher by 471% in the DR/GE group than in the NR/NS group (Figure 9(b)). As for creatinine clearance, it was significantly higher by 240% in the DR/NS group than in the NR/NS group. Conversely, in the DR/GE rats, although the creatinine clearance was still higher by 98% than the level calculated for the NR/NS, it was less by 42% compared to the magnitude estimated for the DR/NS group (Figure 9(c)).

## 4. Discussion

Diabetes mellitus is one of the most morbid medical conditions that aggressively afflict a growing number of the world's population [47]. Type-1 DM results from a reduction in insulin secretion that can vary and accordingly determines the severity of this condition. This form of DM can be induced in rats by chemically destroying their pancreatic insulin-producing *β*-cells using the drug STZ. Experimentally produced STZ-type-1 DM rats develop most of the signature symptoms that are manifested in “naturally” afflicted diabetic humans [48]. It is well known that type-1 DM is a progressive disease that exhibits chronologically varied characteristics dependent on the time of commencement and aggressiveness of the different pathobiochemical-physiological mechanisms. The arguments presented in this section relate to the renal ACE-1 and AngII findings of this study and pertain to a certain transient stage in the life of STZ-induced DM rats.

In this study and at week 8 following the induction of type-1 DM, the DR/NS showed all the expected symptoms of the early stages of the condition. Many of the early typical abnormalities targeted and observed here included severe hypoinsulinemia (Figure 5), hyperglycemia (Figure 1), body weight loss (Figure 2), water intake (Figure 3(b)), urine output (Figure 3(a)), serum albumin decline (Figure 8(a)) with albuminuria (Figure 8(b)), and elevation in serum and urine creatinine concentration and creatinine clearance (Figure 9), in addition to moderate hypertension (Figure 4). Most of these symptoms have been reported previously in a review by Eleazu et al. [49]. The currently observed pathological rise in water, albumin, and creatinine clearance, as indicators of diseased renal functioning, that is, hyperfiltration and reduced tubular reabsorption, can be taken as evidence of nephronal structural remodeling including glomerular and tubular injury, which have been observed in our laboratory as well as others in early DM [37, 41, 50]. This renal injurious structural remodeling, hence refunctioning, could be the result of increased oxidative stress [51], which, in addition to causing many basic structural deformities [8, 16, 20], leads to abnormal receptor expression of advanced glycation end product (AGEs) [52] that could be partly responsible for glomerular glycation [53]. In addition, the augmented renal clearance function could result from a reduction in the intrarenal AngII concentration, and therefore a decline in this octapeptide stimulated absorptive power, as discussed next.

One of the focal objectives of this study was investigating the nature of modulations occurring in the levels of plasma and kidney ACE-1 and AngII in the early stages of STZ-induced DM. The ACE-1 observations of DR/NS (Figure 6) in the current study are in line with the view that ACE-1 concentration increases in the plasma and decreases in the kidney [30]. Furthermore, the levels of AngII show that the changes in this octapeptide concentration are in parallel with those of ACE-1, where the systemically measured ACE-1 and AngII increased simultaneously (Figures 6(a) and 7(a)), while the renally measured ACE-1 and AngII decreased simultaneously (Figures 6(b) and 7(b)). What supports the present study's systemic and renal AngII findings are the following measured physical parameters: first, the detected elevation in BP (Figure 4), which could be the result of a rise in the general vascular resistance induced by an increased vasoactivity caused by increased systematic levels of AngII (Figure 7) [7] and second, the observed tremendous increase in renal clearance of water, albumin, and creatinine (Figures 3, 8, and 9), which could possibly have resulted from a reduction in the renal conservation power of the decreased kidney AngII (Figure 7). This possibility is supported by previous studies by our group and others that showed an increase in the kidney's AT_1_ receptors in DM [16, 44, 54] indicating a reduction in renal AngII concentration and hence its induced biological effects. This form of dynamic reciprocal change between AngII and its AT_1_ receptor represents a classical ligand-receptor relationship that strives to maintain a proper physiological sensitization.

As far as BP is concerned, examination of previous studies carried out on STZ-induced type-1 DM may suggest the existence of two conflicting views. On one hand, a group of studies have reported no change [55] or even a decrease [56, 57] in the exhibited BP. Alternatively, more recent studies, in agreement with the current observation, reported hypertension in the majority [58–60] or a good percentage of the diabetic rats [61]. This view is even supported by the findings of a human study on the same type of diabetes [62]. It is highly conceivable that these differences in the reported BP findings could have been due to the fact that those studies in which observations are inconsistent with the current view were carried out on different rat gender [55], time of measurement and BP model [56], and/or method of measurement [57].

Exploring the GE effect on DM rats' plasma and kidney ACE-1 and AngII concentrations revealed a further dimension to the corrective actions of this natural product. First and as reported here earlier, DR/GE exhibited a significantly higher level of serum insulin (Figure 5), which was associated with more than 50% lower blood glucose (Figure 1), thus providing further support to the suggested hyperinsulinemic-hypoglycemic mechanism of the corrective action of GE reported in the STZ-DM model [37, 39, 41]. Second, and in relationship to another main objective of this study, the corrections in the levels of insulin and glucose by GE were associated with restorations of plasma and renal ACE-1 and AngII concentrations, specifically, a decrease in the plasma AngII concentration associated with a reduction in plasma ACE-1 (Figures 6 and 7) and, at the same time, an increase in renal AngII concentration associated with a rise in renal ACE-1 concentration (Figures 6 and 7). The reduction in the systemic AngII generation correlated nicely to the lower BP measured in response to GE treatment (Figure 4), while the increase in renal AngII availability correlated well with the documented effect of GE on restoring normal AT_1_/AT_2_ balance that is distorted in DM [43, 44]. Furthermore, the increase in renal AngII concentration, in spite of the reported reduction in AT_1_ receptor expression [43], suggests an increase in the antidiuretic processes and a reduction in renal albumin and creatinine clearance, which was observed here in the DR/GE. The reduction in these clearance variables also could have been mediated by the attenuation of oxidative stress processes [50], decreased AGE formation [63], and most importantly the reduction of glomerular glycation [53], in addition to actions which presumably delay the progression of diabetic nephropathy. In this study, the observed changes in the concentration of plasma and kidney ACE-1 and AngII and their effects on BP and renal clearance function are in agreement with earlier reports [5–7, 12].

**Figure 10:**
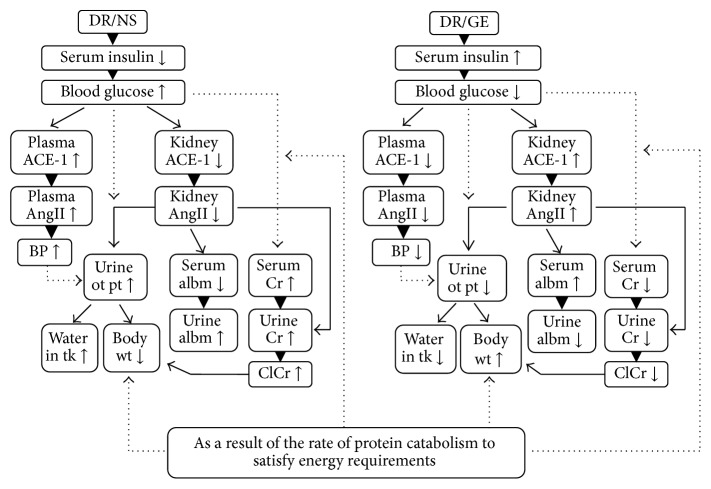
Endocrinal, biochemical, and physiological changes in DR/NS (compared to NR/NS) and DR/GE (compared to DR/NS). The schematic suggests that in early STZ-induced hypoinsulinemia the ensuing hyperglycemia leads to an increase in plasma ACE-1 and, as a result and simultaneously, plasma AngII with a concomitant decrease in kidney ACE-1 and, hence, kidney AngII. As a result of these modulations, BP, water intake, serum creatinine, and renal clearance of water, albumin, and creatinine increased significantly; in addition body weight and serum albumin decreased significantly. Conversely and as seen in the DR/GE group, treatment with GE significantly attenuated and counteracted the modulations observed in the DR/NS. DR/NS: diabetic rats treated with normal saline; DR/GE: diabetic rats treated with garlic extract; ACE-1: angiotensin converting enzyme type-1; AngII: angiotensin II; BP: blood pressure; body wt: body weight; urine ot pt: urine output; serum albm: serum albumin; urine Cr: urine creatinine; water in tk: water intake; ClCr: clearance of creatinine; ↑: increase in concentration or amount; ↓: decrease in concentration or amount; ▼/↓: leads to; straight-lined-arrows: suggestions made depending on the data of this study; dotted-lined-arrows: scientific facts.

In this study, the observed concurrent rise in both serum and urine creatinine concentrations presents a different perspective regarding the diabetic kidney clearance function of this variable in the early stages of type-1 DM. Typically, it is suggested that the diabetic kidney GFR declines as a result of glomerulonephritis leading to a fall in creatinine clearance and consequently a rise in its serum concentration [64]. This sort of creatinine handling materializes towards the late stages of DM and the beginning of renal failure. However, during the early onset of DM and when renal clearance function is exaggerated, a greater clearance of creatinine is expected as observed in this study. In connection, the most plausible interpretation for the current rise in urine creatinine, as suggested by the schematic in Figure 10, is the following metabolic cascade: (1) the development of a higher steady-state rate of protein catabolism as an alternative source of energy [65] to compensate for the decline in cellular uptake of glucose due to hypoinsulinemia [66]. This suggestion is supported by the finding in this study that DR/NS were abnormally lean and showed a continuous pathological decline in body weight compared to NR/NS, as well as DR/GE. As expected and consequent to this elevated protein catabolism is a surge in plasma creatinine load and (2) highly elevated GFR in this early stage of DM. This prediction is supported by the increase in creatinine excretion observed in this study, as well as polyuria and glycosuria reported here and elsewhere [67], all of which are classical symptoms of an early diabetic kidney.

Finally, it is worth stating the following: because DM is a progressive disease, the changes in its physiobiochemical mechanisms, associated with developing structural modifications, are also progressive. Therefore and in the early stages, type-1 DM starts with an increase in renal clearance functions permitted by “appropriate” nephronal and renal mechanistic and structural modifications, which shift to a gradual decline in renal clearance function, again, facilitated by “appropriate” nephronal and renal mechanistic and structural alterations, ultimately leading to the classical end-stage case of renal failure. In support, Kikkawa et al. [68] suggested that hypoinsulinemia-induced hyperglycemia causes a biphasic alteration of RAS due to volume depletion and later due to volume expansion. The control DM rats' data in this study, in particular the renal ACE-1 and AngII changes, support the excretory behavior that is noticed here and is well known to occur at the early stages of type-1 DM. The data of this study also suggest a strong correlation between changes in ACE-1 and AngII and insulin and glucose concentrations in DM rats. A relationship between AngII production and glucose concentration, although opposite to what was observed in this study, was shown by Singh et al. [69] in culture studies. Irrespective of the nature of change in AngII generation, the current observations in DR/GE give further support the view that the GE corrective actions on the ACE-1 and AngII concentration are closely mediated via the insulin-glucose pathway. Accordingly, it is highly probable that, whatever the stage of DM, GE may be effective in slowing down distortion of mechanisms, especially those related to ACE-1 and AngII concentration modulation that affect renal structure and function.

## 5. Conclusion

The findings of this study suggest that the ameliorative action of garlic on the elevated BP and renal clearance functions in early STZ-induced type-1 DM may be partially mediated through attenuating modulations in plasma and renal ACE-1 and AngII concentrations.

## Abbreviations

ACE-1: Angiotensin I converting enzyme type-1
AGE: Advanced glycation end product
AngI: Angiotensin I
AngII: Angiotensin II
AT_1_: Angiotensin type-1 receptor
AT_2_: Angiotensin type-2 receptor
BP: Blood pressure
DM: Diabetes mellitus
DR/GE: Diabetic rats/garlic extract
DR/NS: Diabetic rats/normal saline
GC-MS: Gas chromatography-mass spectroscopy
GE: Garlic extract
GFR: Glomerular filtration rate
LSD: Least significant difference
NR/NS: Normal rats/normal saline
RAS: Renin-angiotensin system
SEM: Standard error mean
STZ: Streptozotocin.
